# Informal Care and Sleep Disturbance Among Caregivers in Paid Work: Longitudinal Analyses From a Large Community-Based Swedish Cohort Study

**DOI:** 10.1093/sleep/zsx198

**Published:** 2017-12-08

**Authors:** Lawrence B Sacco, Constanze Leineweber, Loretta G Platts

**Affiliations:** 1 Department of Global Health and Social Medicine, Institute of Gerontology, King’s College London, London, UK; 2 Stress Research Institute, Stockholm University, Stockholm, Sweden

**Keywords:** carer, cohort study, epidemiology, informal care, Karolinska Sleep Questionnaire, SLOSH, sleep disturbance

## Abstract

**Study Objectives:**

To examine cross-sectionally and prospectively whether informal caregiving is related to sleep disturbance among caregivers in paid work.

**Methods:**

Participants (*N* = 21604) in paid work from the Swedish Longitudinal Occupational Survey of Health. Sleeping problems were measured with a validated scale of sleep disturbance (Karolinska Sleep Questionnaire). Random-effects modeling was used to examine the cross-sectional association between informal caregiving (self-reports: none, up to 5 hours per week, over 5 hours per week) and sleep disturbance. Potential sociodemographic and health confounders were controlled for and interactions between caregiving and gender included. Longitudinal random-effects modeling of the effects of changes in reported informal caregiving upon sleep disturbance and change in sleep disturbance was performed.

**Results:**

In multivariate analyses controlling for sociodemographics, health factors, and work hours, informal caregiving was associated cross-sectionally with sleep disturbance in a dose–response relationship (compared with no caregiving, up to 5 hours of caregiving: β = 0.03; 95% CI: 0.01, 0.06, and over 5 hours: β = 0.08; 95% CI: 0.02, 0.13), results which varied by gender. Cessation of caregiving was associated with reductions in sleep disturbance (β = −0.08; 95% CI: −0.13, −0.04).

**Conclusions:**

This study provides evidence for a causal association of provision of informal care upon self-reported sleep disturbance. Even low-intensity care provision was related to sleep disturbance among this sample of carers in paid work. The results highlight the importance of addressing sleep disturbance in caregivers.

**Statement of Significance**

Sleep disturbances are a common health problem. One likely cause of sleep disturbance is provision of informal care to an elderly, ill, or disabled person. In this prospective analysis of 21 604 participants in paid work, caregiving was associated with self-reported sleep disturbance, particularly at higher numbers of hours of informal care. The effects of providing informal care at different intensities varied between women and men. In analyses of change in caregiving, providing caregiving at the first but not the second wave was associated with reduction in sleep disturbance. These findings support the hypothesis that provision of informal care affects the carer’s sleep quality. Further large-scale studies to examine the mechanisms of sleep disturbance among in-work carers are required.

## INTRODUCTION

Sleeping problems are common: in Swedish adults aged 18–84, insomnia disorder, that is, insomnia symptoms and daytime consequences, has an estimated prevalence of around 10.5% and symptoms of insomnia are reported by one-quarter of the population.^1^ Evidence is growing that people with insomnia symptoms have a raised risk of developing physical illness,^2^ specifically cardiometabolic disease, occupational injuries, and all-cause mortality.^3,4^ In terms of mental illness, disturbed sleep and tiredness are common symptoms of mood disorders such as depression.^5^ Consequently, addressing the causes of insomnia may contribute to improvements in population health.^6^

One likely important cause of sleep disturbance is provision of informal care to an elderly, ill, or disabled person. Informal care is assistance provided by people from the intimate environment of the dependent person, who do not receive any training or economic compensation. It is common: in the United States, 16.6% of adults^7^ and in Sweden, 10.9% of adults^8^ are currently informal carers, rates that are expected to increase in line with population aging and cut-backs in publicly funded care.^9^ Informal caregiving has been associated, albeit inconsistently, with a range of poor health outcomes among carers in large cross-sectional and prospective studies,^10–13^ depending on the characteristics of the carer and person cared for, type of health outcome, type of care provided, and duration of follow-up.^14,15^ However, some studies find that caregiving may be neutral or benefit carers’ health, e.g., by providing an additional rewarding role (*role enhancement*) and improving the quality of family relationships.^16–20^ Among other factors, it is probable that the intensity and duration of care provision determine whether and to what degree caring has a positive or negative impact on the caregiver.

Although it has been argued that various aspects of the caregiving role impact on sleep specifically,^21^ recent reviews of the evidence note that few adequately powered, community-based, longitudinal studies have examined the impacts of caregiving on sleep.^22–24^ Existing studies point to poorer sleep among caregivers, but apart from certain exceptions, tend to be cross-sectional, use small samples which make effects hard to detect, or only examine specific groups of carers without comparing them to the general population. Important confounders such as the caregiver’s education level or health may not be controlled for, factors which lead to both selection into the caregiving role and sleep disturbance.^17^ Stronger evidence would also be provided by prospective designs linking changing provision of care to changes in sleep, but almost no research has so far done so.^25,26^

Although recent reviews have argued for greater focus on subpopulations of carers in terms of factors such as age,^27^ or care recipient characteristics,^28^ to the best of our knowledge, prior research has not examined the impact of caregiving on sleep in the important and large population of caregivers who are simultaneously engaged in paid work. This is despite evidence that difficulties combining work tasks with family obligations can generate stress and health problems.^29,30^ Specifically, the demands of paid work may conflict with requirements to provide informal care, which may lead to *role overload*, in which an individual with a finite amount of time and energy experiences strain from trying to fulfil multiple roles.^16^

Consequently, this study focuses on caregivers and noncaregivers who are in paid work, and uses a large community-based prospective dataset to examine both the cross-sectional and longitudinal associations of caregiving with sleep disturbance. Important health and demographic confounders of the caregiving-sleep relationship are controlled for. Firstly, we examine whether greater intensity of caring, operationalized using weekly hours of care provided, is associated with sleep disturbance in cross-sectional analyses. We hypothesize that providing informal care will be associated with sleep disturbance, although the benefits of “role enhancement” may mean that carers providing low-intensity caregiving experience lower levels of sleep disturbance than noncaregivers. Since women tend to provide more informal care than men,^7^ and reports of insomnia symptoms are more frequent for women than for men,^1^ gender interactions are included in these analyses in case the relationship between caregiving and sleep disturbance differs by gender. Secondly, in longitudinal analyses, we examine the effects of changes in caregiving upon sleep disturbance and upon change in sleep disturbance. We hypothesize that commencing informal caregiving will be associated with greater sleep disturbance and cessation of caregiving with reduced sleep disturbance. To the best of our knowledge, only one study has previously examined the relationship between starting and stopping provision of informal care on sleeping problems, in a small sample of spousal Alzheimer caregivers.^26^ In short, the longitudinal analyses performed in this study may offer additional evidence for a causal relationship between informal caregiving and sleep disturbance in a large sample of in-work carers.

## METHODS

### Study Population

Participants in the Swedish Longitudinal Occupational Survey of Health (SLOSH) formed the study population. SLOSH is a biennial postal survey, which follows a subsample of gainfully employed people aged 16–64 from the Swedish Labour Force Survey who were recruited into the Swedish Work Environment Surveys (SWES) 2003–2011.^31^ Both SLOSH and the present study have been approved by the Regional Research Ethics Board in Stockholm. All participants provided informed consent.

SLOSH participants respond to one of the two versions of the postal questionnaire: an in-work questionnaire for those currently in paid work at least 30% of full-time and another for those working less or who are outside the labor force whether permanently or temporarily. The present study is based on information provided in the in-work questionnaires only from 2010, 2012, 2014, and 2016. Response rates to the SLOSH questionnaires were 57% in 2010 and 2012, 53% in 2014, and 51% in 2016. In total, 21604 participants responding to at least one in-work questionnaire were included in the study, providing 42928 observations for the analysis. The change analyses required participants to respond to the in-work questionnaire in at least two consecutive waves, a requirement which reduced the sample to 12253 people.

### Sleep Disturbance

Self-reported sleep disturbance was measured at each wave using a validated measure of disturbed sleep composed of four questions from the Karolinska Sleep Questionnaire.^32–34^ The measure contains the core symptoms of insomnia; specifically participants were asked how often they had been disturbed in the previous 3 months by difficulties falling asleep, repeated awakenings with difficulties going back to sleep, premature (final) awakening, or disturbed or restless sleep, with response options ranging from 0 (“never”) to 5 (“always/five times a week”). Item nonresponse rates were low: 1.1% for one missing item, 0.2% for each of two and three missing items, and 1.1% for four missing items. An average sleep disturbance score was calculated for participants who provided responses to at least three of the four items. Change in sleep disturbance from one wave to the next was calculated by subtracting the score in the earlier wave from the score in the next wave.

### Provision of Informal Care

At each study wave, questions about participants’ time use in a typical work week were used to identify how much care in hours participants provided for a relative other than a child or grandchild. Original response categories were 0 hour (85.8% of sample members), 1–5 hours (12.4%), 6–10 hours (1.2%), 11–15 hours (0.3%), and >15 hours (0.4%). The last three categories were collapsed into >5 hours per week since relatively few respondents provided high numbers of hours of informal care. A dichotomized variable of caregiving was used in analyses examining change between consecutive waves: no care-giving and provision of any care (at least 1 hour per week). This generated the combinations: no caregiving at either wave; no caregiving at the first wave, caregiving at the second wave; caregiving at the first wave but not at the second; and caregiving at both waves.

### Covariates

Selection of covariates was performed by drawing directed acyclic graphs based on existing knowledge of factors which might affect both sleep disturbance and possibilities to provide informal care.^35^ The sociodemographic variables of gender, age, marital status, and education were included in the analyses as possible confounders of the relationship between caring and sleep disturbance. Information regarding gender, age, education, and marital status (0 = married/cohabiting and 1 = nonmarried/divorced/widowed/single) was obtained through linkage using individual person numbers to Statistics Sweden’s Longitudinal Individual Data Base (LISA) administrative register. Age was centered at its mean of 50 years. In order to model nonlinear relationships between age and sleep disturbance, squared and cubic functions of age were generated. Highest education level was grouped into the following categories: compulsory schooling; 2 years upper secondary/vocational training or 4 years upper secondary; university or equivalent shorter than 3 years; and at least 3 years of university or equivalent.

Two variables recorded participants’ perceptions of whether their lives were affected by (1) any of a range of chronic conditions and (2) physical pain or discomfort. These variables were included as confounders because such health problems might prevent carers from providing help to relatives and are also likely to increase sleep disturbance.^36–38^ Specifically, participants indicated whether they had any of the following chronic conditions (hypertension, cardiovascular disease, diabetes, rheumatic disorder, musculoskeletal disorder, mental illness, asthma, obstructive pulmonary disease, migraine, physical disability, cancer, and other illness) and in a separate question whether they had experienced pain or discomfort over the previous 3 months (specifically headache, pain in the neck or shoulders, pain in the lower back, or other pain). For both variables, participants could respond: no; yes, but this does not affect their life at all; yes, this affects their life a little; or yes, this affects their life a lot. The two variables were dichotomized such that reports that chronic disease or pain/discomfort affected the participant’s life either a little or a lot were coded as 1, otherwise they were coded as 0.

Self-rated health and reports of depressive symptoms were included in the analyses as both potential confounders and mediators of the relationship between informal caregiving and sleep disturbance. To measure self-rated health, respondents were asked: “How would you rate your general state of health” with the responses: very good, good, neither good nor bad, quite poor, or very poor, a single-item measure of general physical and mental health which predicts mortality.^39,40^ A brief six-item version of the Symptom Checklist Core Depression Scale (SCL-CD_6_) was used to measure depressive symptomatology.^41–43^ Questions in this scale evaluate how much (from 0 = not at all to 4 = very much) participants were troubled by lethargy or low energy, feeling blue, blaming oneself, excessive worrying, feelings of no interest in things, and feeling that everything is an effort. An overall score (0–24) was obtained by summing responses to all items.

Self-rated health and depressive symptoms were considered to be potential confounders of the informal caring–sleep relationship because poor self-rated health and depressive symptoms might prevent carers from providing help to relatives and also be likely to increase sleep disturbance. However, self-rated health and depressive symptoms might also mediate the relationship from informal caregiving to sleep disturbance; i.e., they may be on the causal path. Previous research has highlighted the importance of physical fatigue and tiredness to how people evaluate their self-rated health.^40,44^ Furthermore, fatigue and sleep disturbance are common symptoms of depressive episodes in the ICD-10 classification of mental and behavioral disorders.

Paid work intensity was measured in four categories indicating time spent weekly in paid work created from participants’ reports (<10 hours, 10–19 hours, 20–29 hours, and ≥30 hours). This covariate could be both a confounder or mediator of the informal caring–sleep relationship: a confounder because time in paid work could affect both the number of hours dedicated to informal caregiving and sleep disturbance, and a mediator because hours spent providing informal care could lead to a decrease in hours of paid work, which in turn may affect sleep disturbance if, for example, this generated financial difficulties.^45^

### Statistical Methods

Statistical analysis was carried out in Stata 14.2 (Stata Corporation, College Station, TX, USA). We performed random intercept (random effect) modeling, which allows the use of full information provided by longitudinal data, while accounting for the dependency of repeated measures within individuals.^46^ These models use both the between- and within-subject components of the variability that are present in longitudinal data.

Two main analyses were performed, which both present results from the random intercept models as regression coefficients with 95% confidence intervals. First, cross-sectional associations between intensity of informal caregiving (no caregiving, up to 5 hours, and over 5 hours) and sleep disturbance were examined. The reference group contained participants who provided no informal care. These bivariate associations are presented as Model 1. In Model 2, variables were included that were considered to be potential confounders (sociodemographics, physical pain, and chronic disease) because they might affect both sleep disturbance and possibilities to provide informal care. In Model 3, additional variables (self-rated health, depressive symptoms, and hours in paid work) were included which might be both confounders and mediators of the informal caring–sleep relationship. In other words, in addition to being likely confounders, these factors may also lie on the causal pathway from informal caregiving to sleep disturbance. Consequently, adjusting on them may remove part or all of any effect of informal caregiving on sleep disturbance (overadjustment). Potential interactions between gender and care-giving intensity were also examined (Model 4).

Second, we examined changes in informal caregiving (whether providing any care or not) between two consecutive waves in relation to sleep disturbance at the second of the two waves, as well as in relation to change in sleep disturbance between the waves. All of the changes between the four available waves were simultaneously modeled in random intercept models. Covariates were taken from the second of the two waves. The reference group contained participants providing no informal care in either of the two waves.

## RESULTS

Descriptive and bivariate statistics of the total study sample (*N* = 21604) are presented in Table 1 using information from the baseline survey year for each individual. Although most of the sample (18531 participants) did not report providing informal care at baseline, 2675 participants (12.4%) reported providing informal care up to 5 hours per week, and 398 participants (1.8%) reported higher number of hours of care. Caregivers were more likely to be female, be older, have a lower education level, report that their life was affected by physical pain or discomfort or by chronic illness, report poorer self-rated health, depressive symptoms, and sleep disturbance, and be in paid work under 20 hours per week. In sensitivity analyses (not shown), caregivers had poorer physical and mental health as well as greater sleep disturbance even after controlling for the average older age of caregivers.

**Table 1:** Descriptive Statistics and Bivariate Associations Between Caregiving Status and Other Variables at Baseline (Swedish Longitudinal Occupational Survey of Health, *N* = 21604).

	Percent or mean (*SD*)			
Variables	Full sample	Noncarers	Caregiving ≤ 5 h per week	Caregiving > 5 h per week
All	100.0%	85.8%	12.4%	1.8%
Gender (*p* < .001)				
Men	44.5%	45.5%	39.7%	29.9%
Women	55.6%	54.6%	60.3%	70.1%
Age (*p* < .001)	49.5 (10.4)	48.9 (10.6)	52.8 (8.5)	54.2 (8.5)
Education (*p* = .003)				
Compulsory	11.0%	10.9%	10.7%	15.1%
High school	46.1%	45.8%	48.2%	45.0%
University < 3 y	14.7%	14.7%	14.8%	17.1%
University ≥ 3y	28.3%	28.7%	26.3%	22.9%
Marital status (*p* < .001)				
Cohabiting/married	55.9%	55.1%	61.3%	56.0%
Other	44.1%	44.9%	38.7%	44.0%
Life affected by pain or discomfort (*p* < .001)				
No	41.4%	42.1%	36.6%	40.0%
Yes	58.6%	57.9%	63.4%	60.1%
Life affected by chronic illness (*p* < .001)				
No	56.4%	57.6%	49.6%	47.5%
Yes	43.6%	42.4%	50.4%	52.5%
Self-rated health (*p* < .001)				
Very good	26.6%	27.4%	21.6%	22.4%
Good	53.6%	53.5%	55.0%	51.5%
Neither good nor bad	14.2%	13.9%	16.0%	18.3%
Poor	5.3%	5.0%	7.1%	7.3%
Very poor	.3%	.4%	.3%	.5%
Depressive symptoms (*p* < .001)	5.4 (5.1)	5.2 (5.0)	6.1 (5.4)	6.5 (5.7)
Time spent in paid work weekly (*p* < .001)				
<10 h	9.4%	9.2%	10.4%	12.3%
10–19 h	16.0%	15.7%	17.6%	19.1%
20–29 h	15.3%	14.9%	18.1%	14.8%
≥30 h	59.3%	60.2%	54.0%	53.8%
Sleep disturbance score (*p* < .001)	1.61 (1.05)	1.58 (1.04)	1.79 (1.10)	1.89 (1.21)

*SD* = standard deviation.

*p*-Values for the relationship between caregiving status and other variables were obtained using a chi-square test for categorical variables, and one-way ANOVAs for testing differences among means across caregiving status groups.

In the random effects model, presented in Table 2, informal care provision was associated with sleep disturbance before (Model 1) and after adjustment for possible socioeconomic and health confounders (Model 2). Specifically, after adjustment, compared with participants who were not providing informal care, those who were providing care up to 5 hours per week reported higher levels of sleep disturbance (β = 0.07; 95% CI: 0.04, 0.09), whereas those who were providing over 5 hours of care reported highest levels of sleep disturbance (β = 0.17; 95% CI: 0.11, 0.23). In Model 3, we adjusted for depressive symptoms, self-rated health and hours in paid work, and variables which could be both confounders and mediators of the association between caregiving and sleep. Following this adjustment, caregiving was still associated with higher levels of reported sleep disturbance (up to 5 hours of care: β = 0.03; 95% CI: 0.01, 0.06 and over 5 hours of care: β = 0.08; 95% CI: 0.02, 0.13).

**Table 2:** Sleep Disturbance in Relation to Level of Informal Caregiving (Swedish Longitudinal Occupational Survey of Health, *N* = 21604, Random Effects Models).

	Model 1		Model 2		Model 3		Model 4	
	β	95% CI	β	95% CI	β	95% CI	β	95% CI
Caregiving (ref: not caring)								
Caregiving ≤ 5 h per week	0.10***	(0.08, 0.13)	0.07***	(0.04, 0.09)	0.03**	(0.01, 0.06)	0.01	(−0.03, 0.05)
Caregiving > 5 h per week	0.21***	(0.15, 0.27)	0.17***	(0.11, 0.23)	0.08**	(0.02, 0.13)	0.20***	(0.10, 0.31)
Gender (ref: male)								
Female	—		0.21***	(0.18, 0.23)	0.16***	(0.14, 0.18)	0.16***	(0.14, 0.18)
Gender x caregiving (ref: not caring and male)								
Care ≤ 5 hpw and female	—		—		—		0.03	(−0.01, 0.08)
Care > 5 hpw and female	—		—		—		−0.18**	(−0.30, −0.06)
Age	—		0.01***	(0.01, 0.01)	0.01***	(0.01, 0.01)	0.01***	(0.01, 0.01)
Age-squared	—		−0.00***	(−0.00, −0.00)	−0.00***	(−0.00, −0.00)	−0.00***	(−0.00, −0.00)
Age-cubed	—		−0.00***	(−0.00, −0.00)	−0.00***	(−0.00, −0.00)	−0.00***	(−0.00, −0.00)
Education (ref: compulsory)								
High school	—		−0.01	(−0.05, 0.04)	−0.01	(−0.05; .03)	−0.01	(−0.05, 0.03)
Uni < 3 y	—		0.04	(−0.01, 0.09)	0.04	(−0.01, 0.08)	0.04	(−0.01, 0.08)
Uni ≥ 3 y	—		0.06*	(0.01, 0.10)	0.04*	(0.00, 0.08)	0.04*	(0.00, 0.08)
Marital status (ref: married)								
Other	—		0.02	(−0.00, 0.04)	−0.02	(−0.04, 0.00)	−0.02	(−0.04, 0.00)
Pain (ref: none)								
Yes, life affected	—		0.27***	(0.25, 0.29)	0.13***	(0.11, 0.15)	0.13***	(0.11, 0.15)
Chronic disease (ref: none)								
Yes, life affected	—		0.18***	(0.16, 0.20)	0.06***	(0.04, 0.08)	0.06***	(0.04, 0.08)
Self-rated health (ref: very good)								
Good	—		—		0.17***	(0.15, 0.19)	0.17***	(0.15, 0.19)
Neither good nor bad	—		—		0.38***	(0.35, 0.40)	0.37***	(0.35, 0.40)
Poor	—		—		0.53***	(0.49, 0.57)	0.53***	(0.48, 0.57)
Very poor	—		—		0.68***	(0.54, 0.81)	0.68***	(0.55, 0.81)
Depressive symptoms	—		—		0.07***	(0.07, 0.07)	0.07***	(0.07, 0.07)
Time spent in paid work weekly (ref: ≥30 h)								
<10 h	—		—		0.02	(−0.01, 0.05)	0.02	(−0.01, 0.05)
10–19 h	—		—		−0.01	(−0.03, 0.02)	−0.01	(−0.03, 0.02)
20–29 h	—		—		0.01	(−0.01, 0.03)	0.01	(−0.01, 0.03)
Constant	1.61***	(1.59, 1.62)	1.26***	(1.22, 1.31)	0.91***	(0.87, 0.95)	0.91***	(0.87, 0.95)

CI = confidence interval.

*p < .05; **p < .01; ***p < .001.

**Figure 1:**
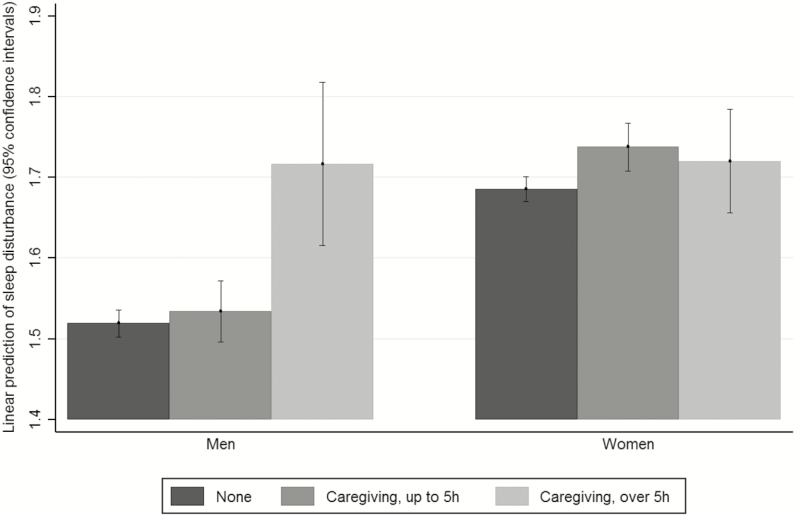
Predictive margins of sleep disturbance in relation to hours per week spent caring and gender (Swedish Longitudinal Occupational Survey of Health, *N* = 21604, random effects models adjusted for socioeconomic and health covariates as well as time in paid work).

Gender interacted significantly with caring (Table 2, Model 4 and Figure 1). Men who did not provide care and who provided up to 5 hours of care had similar levels of sleep disturbance. Both groups had significantly lower sleep disturbance than men who provided over 5 hours of care. Women who provided up to 5 hours of informal care weekly reported slightly more sleep disturbance than women who provided no care; differences between those groups and women providing more than 5 hours of care were not discernible (Figure 1).

In Table 3, random effects models of change in caregiving on both sleep disturbance and change in sleep disturbance are presented. The left-hand set of analyses present change in caregiving from one wave to the next on sleep disturbance at the second of the two waves. Compared with participants who did not provide informal care in either wave, those who provided care during at least one time point had greater sleep disturbance both before (Model 1) and after adjustment (Model 2) for a range of possible confounders at the current wave. Specifically, compared with participants who did not provide informal care in either wave, those who provided care at both waves had significantly greater sleep disturbance (β = 0.10; 95% CI: 0.04, 0.15), as did those who provided care only at the second of the two waves (β = 0.07; 95% CI: 0.03, 0.12), and those who provided care at the first, but not at the second wave (β = 0.05; 95% CI: 0.00, 0.10). Results from Wald tests showed that differences in sleep disturbance among these three categories in which participants provided care in at least one wave were not significant at the 5% level. Only the coefficient for high sleep disturbance for those who were caring in both waves remained significant at the 95% level after including variables in Model 3 (self-rated health, depressive symptoms, and time spent in paid work), which may be both confounders and mediators of the caregiving–sleep relationship.

**Table 3:** Sleep Disturbance in Relation to Changes in Informal Caregiving (Swedish Longitudinal Occupational Survey of Health, *N* = 12253, Random Effects Models).

	Caregiving at t_–1_ and t_1_ on sleep disturbance at t_1_, β (CI)^a^			Caregiving at t_−1_ and t_1_ on change in sleep disturbance from t_−1_ to t_1_, β (CI)^b^		
	Model 1	Model 2	Model 3	Model 1	Model 2	Model 3
Caregiving (ref: no caregiving at either t_−1_ or t_1_)						
No caregiving at t_−1_, caregiving at t_1_	0.13*** (0.08, 0.18)	0.07** (0.03, 0.12)	0.03 (−0.01, 0.08)	0.03 (−0.02, 0.08)	0.02 (−0.03, 0.07)	0.01 (−0.04, 0.06)
Caregiving at t_−1_, no caregiving at t_1_	0.10*** (0.05, 0.14)	0.05* (0.00, 0.10)	0.02 (−0.02, 0.06)	−0.07** (−0.11, −0.02)	−0.07** (−0.12, −0.02)	−0.08*** (−0.13, −0.04)
Caregiving at t_−1_ and t_1_	0.18*** (0.12, 0.23)	0.10*** (0.04, 0.15)	0.05* (0.00, 0.09)	0.00 (−0.04, 0.04)	−0.00 (−0.05, 0.04)	−0.02 (−0.06, 0.02)

CI = confidence interval.

*p < .05; **p < .01; ***p < .001.

Model 1 was unadjusted.

Model 2 included gender, age, age-squared, age-cubed, education level, marital status, pain, and chronic disease at the second of the two waves.

Model 3 additionally contained self-rated health, depressive symptoms, and hours in paid work at the second of the two waves.

^*a*^The outcome is sleep disturbance at t_1_.

^*b*^The outcome is change in sleep disturbance between t_−1_ and t_1_.

The right-hand set of analyses present change in caregiving from one wave to the next upon change in sleep disturbance over the same period. Compared with participants who did not provide informal care in either wave, providing caregiving at the previous but not the current wave was associated with a reduction in sleep disturbance over the same time period, results which changed little after adjustment for possible confounders and mediators (Model 2: β = −0.07; 95% CI: −0.12, −0.02 and Model 3: β = −0.08; 95% CI: −0.13, −0.04). Results from Wald tests showed that this group who provided care only at the first wave also reported a reduction in sleep disturbance in comparison to the other two caregiving groups ( compared with caregiving only at the second wave: χ^2^: 7.85, *p* = .005; compared with caregiving at both waves: χ^2^: 4.45, *p* = .035). Performing caregiving in both waves and providing care only at the current wave were not associated with change in sleep disturbance compared with the reference group composed of participants who did not provide care at either wave.

## DISCUSSION

In this large sample of Swedish people in paid work, providing informal care predicted higher levels of self-reported sleep disturbance, particularly when more than 5 hours of care per week were provided, associations which were robust to inclusion of demographic and health covariates as well as time in paid work. These results correspond to previous research suggesting that informal caregiving impacts on self-reported and objective sleep^22–24,47^ and that providing informal care for a higher number of hours is more strongly associated with sleep disturbance.^48,49^ Studies have shown that provision of nighttime care is associated with poorer self-reported sleep,^49^ as is (albeit inconsistently) when carers and care recipients are coresident.^50–52^ It might be expected that providing relatively low intensity of informal care (up to 5 hours a week) would be associated with better sleep through the mechanism of role enhancement. However, in this Swedish working sample, even low intensity caregiving was associated with self-reported sleep disturbance, suggesting that in-work carers may be experiencing stress generated by role overload in managing the competing commitments of paid work and informal caregiving.

In terms of the stress process model, in which primary stressors generated by informal caregiving lead to problems in other domains, such as job-caregiving conflicts,^53^ such role overload may be generated due to the difficulty of apportioning time and energy to two relatively inflexible activities.^54^ In-work carers have partial control at best over the timing of caregiving and paid work activities, leading to scheduling difficulties (e.g., providing care at unpredictable times, at night, and during the working week), and the difficulty of catching up on sleep during the day due to the fixed timing of most jobs. Informal caregiving, unlike other unremunerated activities, is often performed out of a sense of moral obligation, particularly when the care recipient is a close family member, leaving some caregivers with little or no choice about engaging in caregiving.^55^ The impacts of caregiving upon sleep disturbance extend beyond the time taken for care tasks: caregivers may be on 24-hour call^21,56^ and the emotional labor involved in caregiving can generate substantial worry and distress. Accordingly, introduction of depressive symptoms and self-rated health into the models attenuated the relationship between provision of informal care and sleep disturbance. This result accords with prior research which has highlighted the comorbidity of insomnia symptoms with depression^24^ and self-rated health,^57^ and found cross-sectional and longitudinal associations between sleep and affect in caregivers.^25,58,59^

In contrast to a previous study which failed to find gender differences in the impact of caregiving upon sleeping problems,^50^ we found significant interactions between gender and informal caring in relation to sleep disturbance, specifically similar levels of sleep disturbance in men providing no caregiving and up to 5 hours of caregiving and much higher sleep disturbance among men providing more than 5 hours of care. Women had higher levels of sleep disturbance overall; differences among women by caregiving status were smaller than among men. It is possible that gender differences in sleep disturbance at moderate levels of caregiving are due to different tasks being performed by male and female carers, e.g., more personal care and routine tasks being performed by female carers.^60,61^ Higher intensity caregiving is more often spousal care, where gender differences in care tasks are smaller.^60^ It would be valuable for future research to examine gender differences in large samples, where information about the carer burden, who is cared for and the tasks performed, is available, in order to examine possible mechanisms generating gendered differences in the impact of caregiving upon sleep disturbance.

Relatively little prior research has examined the relationship between starting and stopping provision of informal care and sleep disturbance, an approach which provides additional evidence for the direction of effects. We found that, compared with not providing informal care in either wave, patterns of caregiving between two study waves that would correspond to beginning, terminating, or continuing to provide informal care were associated with higher sleep disturbance at the second of those study waves. The finding that reporting providing care only at the first wave was associated with sleep disturbance at the second wave is in line with qualitative research suggesting that sleep disturbance extends beyond the end of informal care provision.^62^

In analyses relating changes in sleep disturbance from one wave to the next to patterns of caregiving over the same period, individuals who provided informal care only at the first wave (which would correspond to ceasing caregiving) experienced improved self-reported sleep compared with the reference group of participants not providing informal care at either wave. These results point to cessation of caregiving generating reductions in sleep disturbance, even if not to the level of those who had not provided informal care at either wave. They stand in contrast to the sole earlier study examining transitions out of caregiving, which used a small sample of spousal Alzheimer caregivers and differentiated caregiving ceasing as a result of spousal institutionalization or death.^26^ That study, by von Känel et al., found no impact on self-reported or objective sleep measures of spousal institutionalization and a negative impact of spousal death on nighttime wakening and sleep time (measured with actigraph data), but no effect on self-reported sleep complaints.

### Strengths and Limitations

The major strengths of this study are its use of a validated measure of sleep disturbance with good measurement properties in a longitudinal analysis of a large sample with a wide range of demographic and health covariates. In addition, the random effects model is a robust approach to dealing with unbalanced panels and missing data due to attrition, which is generally found in longitudinal analyses. Rather than having to assume that data are missing completely at random, random effects models make the less restrictive missing at random assumption, i.e., the propensity for data to be missing is not related to the missing data after accounting for relationships with observed data.^63–65^ However, the study has certain limitations: first, although the original sample of the SLOSH study was drawn from a representative sample of the Swedish population within the age-range 16–64 years (i.e., the Swedish Labour Force Survey), the findings are not generalizable to those working less than 30% of full-time or outside this age range. In addition, selective attrition from the SLOSH study has taken place, with SLOSH respondents more likely than nonrespondents to be older, female, married, Swedish-born, with university qualifications, and to work in the government sector. Second, it is possible that individuals with disturbed sleep are less likely to provide informal care, as a result of the impact of health conditions associated with disturbed sleep (e.g., depression and chronic pain). Although this would have a conservative effect on the results, weakening the caregiving–sleep disturbance associations, such reverse causation has been reduced by taking such health variables into account. Third, although we were able to control for a wide range of demographic and health variables, there may be residual confounding related to unobserved characteristics which could generate a spurious positive association between participation in informal care and sleep disturbance. Consequently, we performed a sensitivity analysis using fixed effect modeling, which partials out unobserved time-invariant individual differences as well as baseline differences in sleep disturbance by utilizing only information on changes in dependent and independent variables within individuals. The results are presented in Supplementary Table S1 and confirm the conclusions presented in the main analyses. Fourth, measures of care-giving were only provided at each wave; therefore, it was not possible to ascertain what was happening between the waves, e.g., whether a carer providing care at two consecutive waves had been providing care continuously during the 2-year period between those waves. Last, it was only possible to examine associations with weekly hours of care provision because the SLOSH study lacks information about the nature of tasks performed and characteristics of the care recipient (e.g., whether co-resident, nature of disability, or illness), an important avenue for future research in large, community-based samples.

### Concluding Remarks

This study has found that among workers, providing informal care is an independent predictor of sleep disturbance after controlling for demographic and health variables. Even low-intensity caregiving, measured in hours per week, was associated with self-reported sleep disturbance. Sleep disturbance among carers is an under-recognized and under-treated problem,^24^ which is related to a variety of serious health outcomes, including major depressive disorder and physical health complaints. The data are from Sweden, a country with a welfare model aiming to minimize conflict between paid work and caring commitments among informal carers, although recent years have seen cuts to formal care services.^66^ A promising avenue for further research may be to investigate the caregiving–sleep disturbance association in diverse welfare and social care contexts.^67^ That a caregiving–sleep association was observed in a country with relatively comprehensive formal care provision suggests the need for policy actors everywhere to consider measures to support employed caregivers, concerning both government and employers, as well as to protect formal care services. Since it appears that informal carers are at a high risk of sleep disturbance, improved recognition and management by healthcare staff of sleeping problems in carers may also improve carers’ health and quality of life.

## FUNDING

LBS was supported by the British cross-research council Lifelong Health and Wellbeing (LLHW) program under Extending Working Lives as part of an interdisciplinary consortium on Wellbeing, Health, Retirement, and the Lifecourse (WHERL) (ES/L002825/1). LGP received funding from the Swedish Council for Working Life and Social Research (FAS, 2012-1743). SLOSH was supported by FAS (2005-0734) and the Swedish Research Council (VR, 2009-6192 and 2013-1645). The work was carried out within the framework of the Stockholm Stress Center, a FORTE Centre of Excellence (FORTE, 2009-1758).

## DISCLOSURE STATEMENT

*None declared*.

## WORK PERFORMED

Stress Research Institute, Stockholm University, Stockholm, Sweden

## Supplementary Material

Supplementary Table S1
