# Supplementary material for: Informal Care and Sleep Disturbance Among Caregivers in Paid Work: Longitudinal Analyses From a Large Community-Based Swedish Cohort Study
Source: Sleep. 2017 Dec 8;41(2):zsx198. doi: 10.1093/sleep/zsx198 (PMC6018987; doi:10.1093/sleep/zsx198)
Supplement: Supplementary Table S1 [file zsx198_suppl_supplementary-table_s1.docx]

Table S1: Sleep disturbance in relation to level of informal caregiving (Swedish Longitudinal Occupational Survey of Health, *N* = 21 604, fixed effect models)

|  | **β (CI)** | | |
| --- | --- | --- | --- |
|  | **Model 1** | **Model 2** | **Model 3** |
| *Caregiving (ref: not caregiving)* |  |  |  |
| Caregiving ≤ 5 hours per week | .04* (.01; .07) | .04* (.01; .07) | .03 (–.00; .05) |
| Caregiving > 5 hours per week | .12** (.05; .20) | .12*** (.05; .19) | .07* (.01; .14) |

Note: CI = confidence interval; * = p<0.05, ** = p<0.01, *** = p<0.001. Model 2 included gender, age, age-squared, age-cubed, education level, marital status, pain, chronic disease. Model 3 additionally contained self-rated health, depressive symptoms and time spent in paid work.
